# Binding of Host Factors Influences Internalization and Intracellular Trafficking of *Streptococcus uberis* in Bovine Mammary Epithelial Cells

**DOI:** 10.4061/2010/319192

**Published:** 2010-06-03

**Authors:** Raul A. Almeida, John R. Dunlap, Stephen P. Oliver

**Affiliations:** ^1^Department of Animal Science, The Food Safety Center of Excellence, The University of Tennessee, Knoxville, TN 37996, USA; ^2^Department of Botany, The University of Tennessee, Knoxville, TN 37996, USA

## Abstract

We showed that internalization of *Streptococcus uberis* into bovine mammary epithelial cells occurred through receptor- (RME) and caveolae-mediated endocytosis (CME). We reported also that treatment of *S. uberis* with host proteins including lactoferrin (LF) enhanced its internalization into host cells. Since the underlying mechanism(s) involved in such enhancement was unknown we investigated if preincubation of *S. uberis* with host proteins drives internalization of this pathogen into host cells through CME. Thus, experiments involving coculture of collagen-, fibronectin-, and LF-pretreated *S. uberis* with bovine mammary epithelial cells treated with RME and CME inhibitors were conducted. Results showed that internalization of host proteins-pretreated *S. uberis* into mammary epithelial cells treated with RME inhibitors was higher than that of untreated controls. These results suggest that pretreatment with selected host proteins commits *S. uberis* to CME, thus avoiding intracellular bactericidal mechanisms and allowing its persistence into bovine mammary epithelial cells.

## 1. Introduction

To survive in well-protected host microenvironments, bacterial pathogens have evolved pathogenic strategies aimed to bypass host defenses mechanisms. Adherence to and internalization into host cells are bacterial-induced strategies that allow bacterial pathogens to defeat defense mechanisms functional at mucosal surfaces. However, after internalization, pathogens need to overcome intracellular bacteriostatic/bactericidal mechanisms, such as endosome acidification and endosome-lysosome fusion. Classical and nonclassical intracellular bacterial pathogens have evolved strategies to circumvent and even take advantage of bactericidal mechanisms operating in the host cell cytoplasm. Hence, while some pathogens enter host cells via receptor-mediated endocytosis (RME) and exploit acidic endosomal pH to fully express their virulence factors [1, 2], other pathogens exploit caveolae mediated endocytosis (CME), which is not linked to endosomal acidification or fusion with the lysosome [3–8]. Exploitation of CME to gain access into the host cell has been described for a growing list of pathogens, including *S. uberis *[9]. Binding of host proteins such as extracellular matrix proteins has been associated with enhanced adherence to and internalization into host cells by several pathogens. The mechanism by which adherence and internalization are enhanced seems to occur through a molecular bridge (or sandwich) formed by the pathogen, the host protein, and its corresponding receptor located on the surface of the host cell. In many cases, the host protein interacts directly or indirectly with integrins which in turn orchestrate conformational changes that mediate the internalization of the pathogen into the host cell cytoplasm. A molecular bridge conformed by *S. uberis* adhesion molecule (SUAM), bovine lactoferrin, and a putative (LF) receptor in the membrane of MAC-T cells was recently described [10]. This mechanism postulated as a pathogenic strategy by which *S. uberis* exploits the abundance of LF in bovine mammary gland secretion to increase adherence to and internalization into bovine mammary epithelial cells. The pathway by which pathogens ingress into host cells is of paramount importance for the pathogen's intracellular survival, for example RME means for the invading pathogen to face bactericidal mechanisms such as endosome acidification and endosome-lysosome fusion as compared with CME which does not involve these steps. Research conducted in our laboratory showed that increased internalization of *S. uberis* into host cells occurred upon treatment with extracellular matrix proteins (ECM) or LF [11–13]. To define if the binding of these host proteins enhances internalization of *S. uberis* through CME and therefore, increased chances of survival and intracellular persistence, experiments involving bovine mammary epithelial cells treated with CME and RME inhibitors and *S. uberis* pretreated with collagen, fibronectin, and LF were conducted.

## 2. Materials and Methods

### 2.1. Bacterial Species and Culture Conditions

The* S. uberis *strains UT888 and UT366, isolated originally from cows with mastitis, identified using standard bacteriological identification protocols and characterized by PCR-based DNA fingerprinting as described in [14] were used. For internalization assays, *S. uberis* UT888 and UT366, stored at −80°C in 10% skin milk, were thawed in a 37°C water bath, plated onto trypticase soy agar plate supplemented with 5% defibrinated sheep blood (BAP, Becton Dickinson and Company, Franklin Lakes, NJ, USA), and incubated for 16 hours at 37°C. After incubation, bacterial lawns were harvested, resuspended in 20 mL Todd Hewitt broth (THB, Becton Dickinson Co., Sparks, MD), and incubated with orbital shaking (150 rpm) for 2 hours at 37°C (C24 Incubator Shaking, New Brunswick Scientific, Eden, NJ, USA). Bacterial suspensions were then washed three times by centrifugation (2,500 xg, 15 minutes at 4°C) in phosphate buffer saline (PBS, pH 74), resuspended to original volume in PBS (pH 7.4), and diluted 1 : 100 in Dulbecco's Modified Eagle's Medium (DMEM, Gibco, Grand Island, NY, USA).

### 2.2. Mammary Epithelial Cell Culture

A bovine mammary epithelial cell line (MAC-T) was used [15]. MAC-T cells were grown in 24-well cell culture plates (Corning Inc., Corning, NY, USA) or 8-well slides (Lab-Tek II, Nalge Nunc International Corp., Naperville, IL, USA) at 37°C in 5% CO_2_ : 95% air (vol/vol) using a cell growth media (CGM) as described in [9] at a cell density of ~1 × 10^6^ cells/mL. For transcytosis experiments, MAC-T cells were seeded onto 1.0 *μ*m pore-size fibrillar collagen-coated inserts (BD BioCoat Multiwell Insert System, Becton Dickinson, Franklin Lakes, NJ, USA) allowing cells to reach confluence. MAC-T cells monolayer integrity and functional barrier were assessed as described in [16, 17].

### 2.3. Bacterial Internalization Assay

Internalization assays were performed following previously described protocols [9, 11] with modifications. Briefly,* S. uberis* strains UT888 and UT366 were cocultured with MAC-T cells in DMEM (Gibco BRL) with and without addition of fibronectin (FN, 10 *μ*g/mL), collagen (Coll, 25 *μ*g/mL), or LF (1 mg/mL) for 2 hours at 37°C in 5% CO_2_ : 95% air (vol/vol). After removing CGM from MAC-T monolayers, 1 mL of DMEM containing *S. uberis* UT888 or UT 366 (~10^7^ colony forming units per mL (cfu/mL)) was added per well at an MOI of 10, using 3 wells for each strain and condition studied. After incubation (2 hours, 37°C in 5% CO_2_ : 95% air (vol/vol)), monolayers were washed 3 times with PBS (pH 7.4) and incubated with CGM containing gentamicin (100 *μ*g/mL; Sigma Chemical Co., St. Louis, MO, USA) and penicillin G (100 UI, Sigma Chemical Co.) for 2 hours at 37°C in 5% CO_2_ : 95% air (vol/vol). After removal of media containing antibiotics, MAC-T monolayers were washed three times with PBS (pH 7.4), treated with trypsin (0.25% in double distilled H_2_O, Sigma Chemical Co.), and lysed with Triton X-100 (0.025% in sterile double distilled H_2_O, Sigma Chemical Co.). Colony-forming units/mL of internalized bacteria were calculated using standard plate counting techniques. To inhibit CME, MAC-T cells were pretreated with methyl-*β*-cyclodextrin (M*β*C, 10 mM in DMEM, Sigma Chemical Co.) for 1 hour at 37°C in 5% CO_2_ : 95% air (vol/vol). To inhibit RME, MAC-T cells were treated with monodansylcadaverine (MDC, 3 mM in DMEM, Sigma Chemical Co.) for 1 hour at 37°C. For transcytosis experiments, host proteins- treated or -untreated *S. uberis* were cocultured with MAC-T cells pretreated with CME or RME inhibitors, as described above. Transcytosis of *S. uberis* through bovine mammary epithelial cells was assessed by sampling culture medium in the lower compartment of the invasion chamber at 60, 120, and 180 minutes of incubation. Colony-forming units were calculated using standard plate counts techniques. The innocuity of treatments with M*β*C or MDC on bovine mammary epithelial cells was assessed by staining MAC-T cell monolayers with Trypan blue staining and 4′-6-Diamidino-2-phenylindole (DAPI, Invitrogen, Boston, MA, USA) stain.

### 2.4. Scanning Electron Microscopy

MAC-T cell monolayers grown on collagen-coated inserts (BD BioCoat Multiwell Insert System, Becton Dickinson) were cocultured with *S. uberis* in DMEM (Gibco BRL) with and without addition of FN, (10 *μ*g/mL, Sigma Chemical Co.), Coll, (25 *μ*g/mL, Sigma Chemical Co.), or lactoferrin (LF, 10 mg/mL, Sigma Chemical Co.), for 2 hours at 37°C in 5% CO_2_ : 95% air (vol/vol). Monolayers were washed 3 times with PBS (pH 7.4) and fixed by incubation with 2.5% glutaraldehyde in distilled water for 2 hours at 4°C. Subsequently, monolayers were washed with distilled water, postfixed in 1% osmium tetraoxide (Sigma Chemical Co.), and dehydrated through a graded alcohol series. Finally, monolayers were critical point dried, mounted on specimen stub with silver paste, coated with palladium alloy, and examined by SEM (Carl Zeiss SMT Inc., Peabody, MA, USA).

### 2.5. Statistics

Each assay was performed in three independent tests running each experimental condition in triplicate. Means from each experiment were analyzed by ANOVA. Means of experimental conditions that showed statistically significant differences (*P* < .05) were further analyzed by Student's *t* test using ProStat (Poly Software International, Salt Lake City, UT, USA) statistical software.

## 3. Results

### 3.1. Internalization of *S. uberis* Treated with Host Proteins into MAC-T Cells Treated with RME Inhibitors

**Figure 1:**
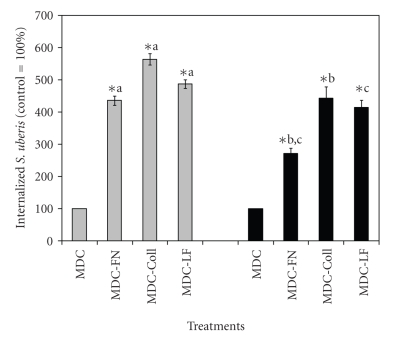
Effect of treatment with host proteins on caveloae-mediated internalization of *Streptococcus uberis* into bovine mammary epithelial cells. Fibronectin (FN), collagen (Coll), and bovine lactoferrin (LF), pretreated *S. uberis* UT888 (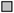) and UT366 (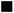) were cocultured with bovine mammary epithelial cells treated with the receptor-mediated endocytosis inhibitor monodansylcadaverine (MDC) and colony forming units per ml (cfu/mL) of intracellular *S. uberis* calculated. Data are presented as the percentage of untreated controls (100%; UT888 = 7.9 × 10^4^; UT366 = 7.0 × 10^3^) and bars represent the standard error of the mean (SEM) of three independent experiments run in triplicate. (∗) indicates statistically significant differences (*P* < .05) with the corresponding control. Means with similar superscript letters within each strain are statistically different (*P* < .05).

Previous research [11, 13] showed that treatment with Coll, FN, and bovine LF enhanced adherence to and internalization of *S. uberis* into bovine mammary epithelial cells. Since it was found that internalization of *S. uberis* into bovine mammary epithelial cells occurred via RME or CME [9], RME was inhibited in bovine mammary epithelial cells with MDC to define if treatment of *S. uberis *with host proteins (Coll, FN, and LF) enhanced internalization into host cells through CME. Under these experimental conditions, internalization of *S. uberis* into host cells via RME would be inhibited and entry into host cells would proceed mainly through CME. Results showed that internalization of *S. uberis *strains pretreated with FN, Coll, and LF into MAC-T cells treated with MDC was significantly higher (*P* < .05) than the corresponding values for untreated *S. uberis *(Figure 1). When the effect of these host proteins on internalization of *S. uberis* into host cells treated with MDC was compared, it was found that treatment of both *S. uberis* strains with Coll induced the highest (*P* < .05) internalization. Treatment of *S. uberis* UT888 with FN, Coll, and LF induced relative higher internalization into host cells than *S. uberis *UT366 under similar treatment conditions.

### 3.2. Internalization of *S. uberis* Treated with Host Proteins into MAC-T Cells Treated with CME Inhibitors

**Figure 2:**
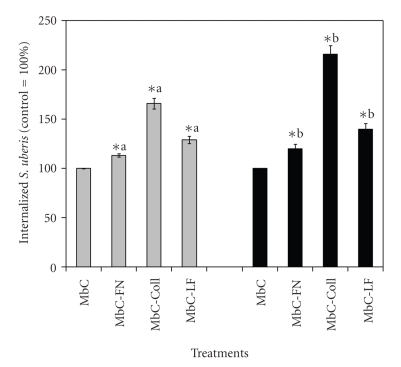
Effect of treatment with host proteins on receptor-mediated internalization of *Streptococcus uberis* into bovine mammary epithelial cells. Fibronectin (FN), collagen (Coll), and bovine lactoferrin (LF), pretreated *S. uberis* UT888 (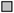) and UT366 (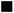) were cocultured with bovine mammary epithelial cells treated with the caveolae-mediated endocytosis inhibitor Methyl-*β*−cyclodextran (M*β*C) and colony forming units per ml (cfu/mL) of intracellular *S. uberis* calculated. Data are presented as the percentage of untreated controls (100%; UT888 = 7.5 × 10^2^; UT366 = 3.0 × 10^2^) and bars represent the standard error of the mean (SEM) of three independent experiments run in triplicate. (∗) indicates statistically significant differences (*P* < .05) with the corresponding control. Means with similar superscript letters within each strain are statistically different (*P* < .05).

Methyl-*β*-cyclodextrin (M*β*C) was used to selectively sequester cholesterol from the plasma membrane, in preference to other membrane lipids, with subsequent loss of caveolae-dependent endocytosis [6, 9, 18]. To elucidate if RME of *S. uberis *into MAC-T cells, which would predominate in eukaryotic cells upon treatment with CME inhibitors such as M*β*C, changed upon treatment of *S. uberis* with host proteins, M*β*C-treated MAC-T cells were cocultured with *S. uberis* pretreated with Coll, FN, and LF. Internalization of *S. uberis* into MAC-T cells treated with M*β*D was less efficient than into MAC-T cells treated with MDC (Figures 1 and 2). Internalization of *S. uberis* pretreated with host proteins into MAC-T cells treated with M*β*D was 1.1 to 1.6 and 1.2 to 2.2 higher than that in untreated controls, whereas the corresponding values for MAC-T cells treated with MDC were 4.4 to 5.6 and 2.7 to 4.4 times higher for the strains UT888 ad UT366, respectively than those for untreated controls. Strain differences were observed. For instance, when internalization of Coll-treated *S. uberis* UT888 and UT366 into MAC-T cells treated with M*β*D was compared, it was found that the strain UT366 was more efficient than the strain UT888.

### 3.3. Transcytosis of ECM-Treated *S. uberis* through MAC-T Cells Treated with RME Inhibitor

**Figure 3:**
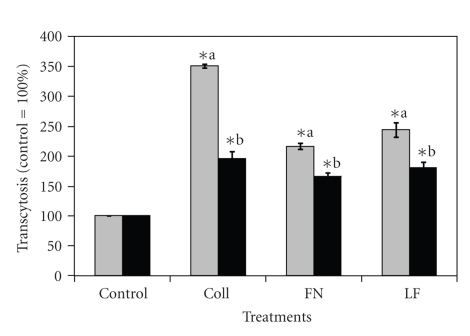
Effect of treatment with host proteins on transcytosis of *Streptococcus uberis* across bovine mammary epithelial cells treated with the receptor mediated endocytosis inhibitor monodansylcadaverine. Fibronectin (FN), collagen (Coll), or bovine lactoferrin (LF), pretreated *S. uberis* UT888 (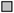) and UT366 (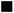) were cocultured with bovine mammary epithelial cells treated with the receptor-mediated endocytosis inhibitor monodansylcadaverine (MDC), and colony forming units per ml (cfu/mL) of *S. uberis* that transcytosed bovine mammary epithelial cells were calculated at 60 minutes. Data are presented as the percentage of untreated controls (100%; UT888 = 6.0 × 10^2^; UT366 = 3.0 × 10^2^) and bars represent the standard error of the mean (SEM) of three independent experiments run in triplicate. (∗) indicates statistically significant differences (*P* < .05) with the corresponding control. Means with similar superscript letters within each strain are statistically different (*P* < .05).

**Figure 4:**
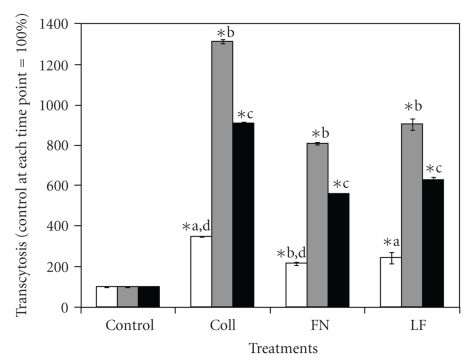
Transcytosis of *Streptococcus uberis* pretreated with host proteins across bovine mammary epithelial cells treated with the receptor mediated endocytosis inhibitor monodansylcadaverine detected at different incubation times. Fibronectin (FN), collagen (Coll), or bovine lactoferrin (LF) pre-treated *S. uberis* UT888 was cocultured with bovine mammary epithelial cells treated with the receptor-mediated endocytosis inhibitor monodansylcadaverine (MDC), and colony forming units per ml (cfu/mL) of *S. uberis* that transcytose bovine mammary epithelial cells were calculated at 60 (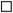), 120 (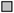), and 180 (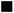) min of incubation. Data are presented as the percentage of transcytosis through monodansylcadaverine treated bovine mammary epithelial cells (100%, 60 minutes = 5.1 × 10^2^, 120 minutes = 6.3 × 10^2^; 180 minutes = 1.0 × 10^3^) at each incubation time and bars represent the standard error of the mean of three independent experiments run in triplicate. (∗) indicates statistically significant differences (*P* < .05) with the corresponding control. Means with similar superscript letters within each sampling time are statistically different (*P* < .05).

Previous data generated by our group showed that *S. uberis* was capable of transcytosing bovine mammary epithelial cells [13]. Reports suggested that transcytosis of bacterial pathogens through host cells could be linked to CME of pathogens into host cells [5, 9]. Since treatment of *S. uberis* with host proteins enhanced the use of CME to internalize into host cells, it was of interest to find if this internalization strategy was linked to increased transcytosis of *S. uberis *through host cells. When MAC-T cells monolayers were treated with MDC inhibitors and then cocultured *S. uberis* pretreated with Coll, FN, and LF, transcytosis was higher as compared with *S. uberis *nontreated controls (Figure 3). When the transcytosis-enhancing effect was compared among host protein treatments, it was found that treatment with Coll resulted in the highest (*P* < .05) transcytosis of *S. uberis* through host cells. Strain differences were also detected; *S. uberis *UT888 showed relative higher transcytosis as compared with *S. uberis *UT366. The effect of host proteins on transcytosis of *S. uberis* across host cells at different incubation times was investigated also. Results presented in Figure 4 show that the highest percentage of transcytosis occurred after 120 minutes as compared to 60 or 180 minutes of incubation.

### 3.4. Scanning Electron Microscopy

**Figure 5:**
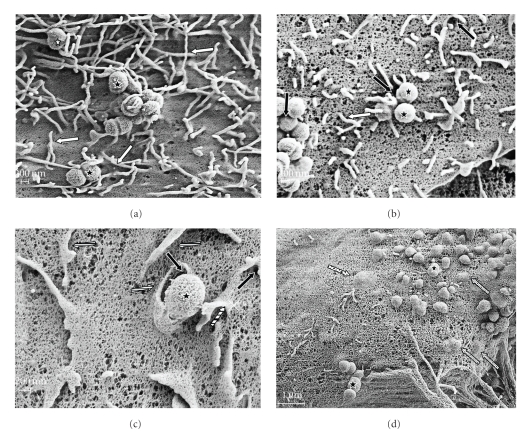
Scanning electron microscopy (SEM) of cocultures of bovine mammary epithelial cells with host-proteins pre-treated *S. uberis*. *Streptococcus uberis* was pre-treated with collagen, fibronectin, or lactoferrin and cocultured with bovine mammary epithelial cells. Cocultures were observed using SEM at different incubation times. Upon contact with *S. uberis *(*) bovine mammary epithelial cells reacted (a) with the formation of plasma membrane invaginations (black arrows) and “spaghetti like” filopodia (white arrows) that stick to and covered (b) *S. uberis* formations of more than one cell. Filopodia then seemed to become flap-like structures (striped arrows) that engulfed single *S. uberis* cells (c) and drove their internalization into host cells through cell membrane invaginations. The internalization process seemed to end with the “patching” of cell membrane invaginations through which *S. uberis* gained access into the host cell (dot-filled arrows (d)) with flap-like structures.

**Figure 6:**
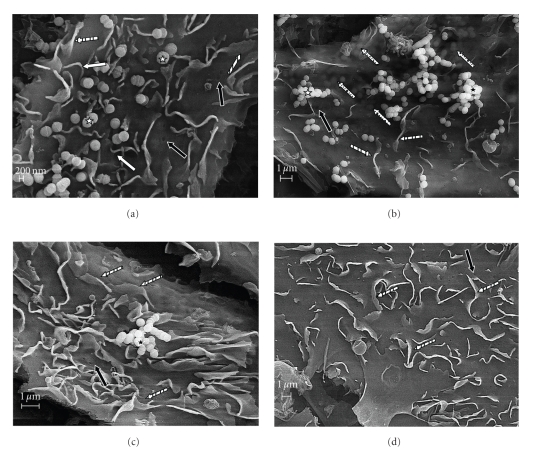
Scanning electron microscopy (SEM) of cocultures of bovine mammary epithelial cells with untreated *S. uberis*. Untreated *Streptococcus uberis* was cocultured with bovine mammary epithelial cells and observed using SEM at different incubation times (a)–(c). Upon contact with *S. uberis *(*) bovine mammary epithelial cells did not reacted abundantly as seen with *S. uberis* pretreated with fibronectin, collagen, or bovine lactoferrin. Although filopodia (white arrows) and flap-like structures (stripped arrows) were detected, these structures were also observed in MAC-T cells not cocultured with *S. uberis* (d). Even though cell membrane invaginations (black arrows) as well as *S. uberis* internalization (dot-filled arrows) were detected, neither flap-like structures engulfing bacteria nor filopodia sticking or covering *S. uberis* were observed.

Scanning electron microscopy inspection of bovine mammary epithelial cells cocultured with *S. uberis *pretreated with host proteins (Coll, FN, and LF) revealed an intense host cell membrane reaction characterized initially by the formation of “spaghetti-like” filopodia at the point of contact with *S. uberis *cells (Figure 5(a)). Upon contact with the plasma membrane, a triggering mechanism seemed to induce the formation of “spaghetti-like” filopodia that stick to and cover *S. uberis* (Figure 5(b)). This process was followed by the formation of ruffles or “flap-like” structures that were detected around plasma membrane invaginations (Figure 5(c)). Internalization of *S. uberis* into host cells appeared to be driven by flap-like structures, which induced internalization of *S. uberis* into host cells through plasma membrane invaginations (Figures 5(c) and 5(d)). In contrast, scanning electron inspection of bovine mammary epithelial cells cocultured with untreated *S. uberis* showed a moderate host cell membrane reaction (Figure 6) as compared with the corresponding for *S. uberis *pre-treated with host proteins. Even though filopodia, flap-like structure, and invaginations were detected, these seemed not to be as involved in internalization of *S. uberis* as when *S. uberis* was pretreated with Coll, FN, or LF (Figure 6(a)–6(c)). Figure 6(d) is an SEM micrograph of MAC-T not cocultured with *S. uberis*.

## 4. Discussion

Binding of host proteins provides bacterial pathogens with important pathogenic advantages such as increased adherence to host cells or masking of surface epitopes, which hamper the function of phagocytic cells [19]. Work from our group showed that when pre-treated with Coll, FN, or LF, *S. uberis* internalized better into bovine mammary epithelial cells than untreated controls [11, 13]. Further research work showed that even though *S. uberis* exploited RME or CME to gain access into host cells, significantly higher internalization occurred through caveloae-mediated mechanisms [9]. Results from the present study showed that internalization of *S. uberis* into bovine mammary epithelial cells treated with an RME inhibitor was enhanced significantly by pretreatment of the pathogen with Coll, FN, or LF. Among these protein treatments, Coll showed the greatest effect on internalization. These results suggest that treatment with these host proteins commits* S. uberis* to internalize into host cells through caveolae-mediated mechanisms. Results from cocultures of similarly pre-treated *S. uberis* with mammary epithelial cells treated with the caveolae inhibitor M*β*D showed that even though internalization was increased, it was significantly lower than when bovine mammary epithelial cells were treated with RME inhibitor. These findings suggest that pre-treatment with Coll, FN, or LF preferentially enhanced internalization of *S. uberis* through the caveolae system than through RME. A common observation from both experiments was that Coll was the host protein treatment that had the greatest effect on internalization of *S. uberis* into host cells.

Previous reports linked caveolae-mediated internalization with transcytosis of pathogens through host cells [20, 21] and research conducted by our group showed that upon pre-treatment of *S. uberis* with LF, transcytosis of *S. uberis *through bovine mammary epithelial cells was higher as compared with untreated controls [13]. Therefore, we investigated if pre-treatment of *S. uberis* with Coll, FN, or LF enhanced transcytosis through bovine mammary epithelial cells treated with the RME inhibitor MDC. Results showed that treatment with these host proteins significantly enhanced transcytosis as compared with nontreated *S. uberis*, suggesting that enhanced transcytosis was a consequence of improved preferential internalization of *S. uberis* into host cells through caveolae-mediated mechanisms.

SEM studies on cocultures of bovine mammary epithelial cells cocultured with *S. uberis *pretreated with Coll, FN, and LF showed a massive bovine mammary epithelial cell reaction characterized by formation of “spaghetti-like” filopodia, flap-like structures, and membrane invaginations occurred at the area where *S. uberis* contacted with the host cell surface. Such changes were much less noticeable in SEM of MAC-T cells cocultured with untreated *S. uberis*.

Entry of ECM or LF treated *S. uberis *into host cells apparently took place through a process driven by flap-like structures that engulfed and internalized *S. uberis *through plasma cell invaginations. This process seems similar to the one described by Molinari et al. [22] in that invaginations through which *S. uberis* internalize into host cell were described in the interaction of *Streptococcus pyogenes* A40, a FN-binding strain, with Hep-2 cells.

Results from SEM of nontreated *S.uberis* showed that bovine mammary epithelial cells did not react with the same intensity as with host proteins-pretreated* S. uberis. *These findings are in agreement with the concept that treatment of *S. uberis* with Coll, FN, or LF enhances entry and facilitates persistence of *S. uberis* inside bovine mammary epithelial cells.

Results presented in this investigation suggest that treatment of *S. uberis* with Coll, FN, or LF enhanced exploitation of caveolae-mediated internalization of this pathogen into host cell and this effect has several important biological implications. Exploitation of CME not only allows *S. uberis* to avoid phagosome acidification and fusion with lysosome but also allows transcytosis of *S. uberis* across host cells, invasion of underlying tissue, and spreading and persistence of *S. uberis* in the mammary parenchyma.

Collagen, FN, and LF and their derivatives are host proteins found in elevated concentrations at the late involution period of bovine mammary glands [23], that is, when, coincidentally, the highest prevalence of *S. uberis* intramammary infections (IMIs) is commonly detected [24]. Very frequently, the infection originated by *S. uberis* during the nonlactating period persists for long periods in the ensuing lactation [14]. Therefore, it is possible that binding of extracellular matrix proteins (Coll, FN, and LF) and their derivatives is a pathogenic strategy that allows *S. uberis* to internalize better into host cells avoiding intracellular bacteriostatic/bactericidal mechanisms and inducing persistent IMI.

In this study, strain differences were found. Results showed that of both *S. uberis* strains pretreated with Coll, FN, or LF, *S. uberis* UT888 internalized into and transcytosed through bovine mammary epithelial cells treated with RME inhibitors better than *S. uberis* UT366, whereas the opposite results were observed when host cells treated with M*β*C were used. Intramammary infections caused by *S. uberis* UT888 are mild, subacute and could progress into chronic persistent infections that can persist for more than one lactation [14]. In contrast, IMIs caused by *S. uberis* UT366 are extremely severe, hyperacute, inducing important systemic clinical symptoms but rarely chronic IMI [25]. Taken together, it can be speculated that the ability of *S. uberis* UT888 to induce chronic infection could be due to its higher capability to exploit caveolae-mediated internalization, which is enhanced by the binding of host factors such as Coll, FN, and LF. With regards to the experimental questions delineated in the introduction, we believe that rather than being an alternative process, binding of host proteins evaluated in this investigation leads to a synergistic process that not only increased internalization but also enhanced intracellular survival of *S. uberis* as compared with untreated controls.

In conclusion, results from this investigation indicate that the binding of Coll, FN, or LF enhanced caveolae-mediated internalization of *S. uberis* into bovine mammary epithelial cells, favoring intracellular survival and persistence of this important mastitis pathogen. Future work should define if a correlation between internalization of *S. uberis* into host cells via caveolae-mediated mechanisms and the ability to induce chronic/persistent IMI exists.
